# Myocardial Work Evaluation—A Useful Non-Invasive Method to Predict Coronary Artery Sub-Occlusion in a Patient with Unstable Angina and Multiple Myocardial Revascularization Interventions

**DOI:** 10.3390/diagnostics13081459

**Published:** 2023-04-18

**Authors:** Alexandru Gheorghiu, Sergiu-Florin Arnautu, Milena Slovenski, Claudiu-Daniel Malița, Mirela-Cleopatra Tomescu, Diana-Aurora Arnautu

**Affiliations:** 1 Multidisciplinary Heart Research Center, “Victor Babes” University of Medicine and Pharmacy, 12 Revolution of 1989 Bd., 300040 Timisoara, Romania; 2 Cardiology Clinic, Timisoara Municipal Clinical Emergency Hospital, 12 Revolution of 1989 Bd., 300040 Timisoara, Romania; 3 Department of Internal Medicine, Victor Babes” University of Medicine and Pharmacy, 2 Eftimie Murgu Sq., 300041 Timisoara, Romania; 4 Department of Radiology and Medical Imaging, Victor Babes University of Medicine and Pharmacy, 300041 Timisoara, Romania; 5 Department of Cardiology, “Victor Babes” University of Medicine and Pharmacy, 2 Eftimie Murgu Sq., 300041 Timisoara, Romania

**Keywords:** coronary artery disease, multi-stenting, in-stent restenosis, native coronary lesions, 2D-speckle echography, global left ventricular strain, myocardial work index

## Abstract

Background: While lifestyle changes, management of coronary artery disease (CAD) risk factors, myocardial revascularization procedures, and medication can improve a patient’s prognosis, de novo native coronary lesions and in-stent restenosis (ISR) remain significant clinical concerns. ISR is more frequent with a bare-metal stent than with a drug-eluting stent and has been documented in around 12% of DES patients. Acute coronary syndrome (ACS) manifests as unstable angina in about 30% to 60% of ISR patients. Myocardial work imaging is a modern, non-invasive technique able to identify individuals with critical coronary artery lesions with high sensitivity and specificity. Case report: We present the case of a 72-year-old Caucasian gentleman with multiple cardiovascular risk factors, admitted to the Cardiology Clinic of Timișoara Municipal Hospital with unstable angina. From 1999 to 2021, the patient experienced two myocardial infarctions, a double aortocoronary bypass graft, and multiple percutaneous coronary interventions with 11 stent implantations, including 6 for ISR. Using two-dimensional speckle-tracking echocardiography and myocardial work assessment, we detected that the lateral wall of the left ventricle had a severely impaired deformation pattern. Angio-coronarography was performed, and sub-occlusion of the posterolateral branch of the right coronary artery was found. Angioplasty was performed and a DES was inserted, with a good final angiographic result and complete release of symptoms. Conclusion: In patients with a history of multiple myocardial revascularization interventions and ISR, it is challenging to identify the critical ischemia region by non-invasive methods. Myocardial work imaging was beneficial in the detection of the altered deformation patterns indicating significant ischemia, its accuracy being superior to that of LV strain, as proven by coronary angiography. Urgent coronary angiography followed by angioplasty and stent implantation resolved the issue.

## 1. Introduction

Cardiovascular disease is still the main cause of mortality worldwide [1], and coronary artery disease (CAD) is a major public health concern [2]. Early identification and treatment are critical in individuals with severe CAD to prevent unfavorable cardiac events such as myocardial infarction, which leads to left ventricular (LV) dysfunction. While lifestyle changes, management of CAD risk factors, myocardial revascularization procedures, and medication can improve a patient’s prognosis, de novo native coronary lesions and in-stent restenosis (ISR) remain significant clinical concerns [3].

In patients with a history of multiple myocardial revascularization interventions and ISR, it is difficult to differentiate de novo native coronary lesions from ISR. Coronary artery angiography detects both types of lesions. ISR is defined as a stent segment or its margins with a ≥50% stenosis of the coronary artery diameter (at the stent segment or 5-mm proximal and distal to the stent) [4,5]. The incidence of ISR with a bare-metal stent (BMS) is higher than with a drug-eluting stent (DES). This is because neointimal growth occurs more commonly after BMS insertion. Restenosis has been documented in around 12% of DES patients. Due to late stent thrombosis, it is more frequent with the use of a first-generation DES. Another degenerative process that contributes to stent stenosis is neo-atherosclerosis. The use of DES has resulted in a considerable reduction in the overall occurrence of ISR. As a result, a DES is both more secure and efficient than a BMS. ISR therapy and management are currently complex and clinically challenging [5].

Myocardial work (MW) imaging using two-dimensional speckle-tracking echocardiography (2D-STE) is a powerful method for assessing myocardial function beyond LVEF and allows for more sensitive detection of localized wall motion disorders [6,7]. It evaluates myocardial deformation through strain interpretation in connection to a dynamic non-invasive LV systolic pressure measurement [8,9,10]. A recent study found that individuals with acute coronary artery obstruction could be identified with high sensitivity and specificity by decreased MW indexes, which are unaffected by LV afterload [8].

We present the case of a patient with multi-vessel coronary artery disease, multiple ISR, and a double bypass coronary graft with normal LV function, in whom decreased myocardial work indexes suggested a new significant coronary artery lesion.

## 2. Case Report

We present the case of a 72-year-old Caucasian male with multivessel coronary artery disease who was admitted to the Cardiology Clinic of the Timisoara Municipal Clinical Emergency Hospital for aggravated rest angina episodes lasting for 10–15 min, recurrent during the last 24 h, and partially relieved by sublingual nitroglycerin. We diagnosed an acute coronary syndrome (ACS) of unstable angina type in a patient with a long history of well-controlled grade 3 hypertension, mild hypercholesterolemia, significant cigarette smoking (60 pack years), peripheral artery disease, and Wolff-Parkinson-White syndrome with recurrent orthodromic re-entry tachycardias.

The patient also has a history of two previous myocardial infarctions: anterior (1999), when thrombolysis with streptokinase was performed, followed by triple bare metal stenting on the left anterior descendent artery (LAD), and inferior (2021), when primary percutaneous coronary intervention (PCI) was performed with double drug-eluting stenting of the right coronary artery (RCA).

The patient has also had bilateral arteriopathy obliterans of the lower limbs, stage IIA, with intermittent claudication at 500 m and paclitaxel stent implantation in the left (1999) and right (2000) superficial femoral arteries.

A coronary angiography performed in 2000 revealed proximal stent restenosis and middle stent occlusion on the LAD, as well as 50–75% stenosis in the middle segment of the RCA, followed by a double aortocoronary bypass graft on the LAD and RCA, using left and right internal mammary arteries.

From 2004 to 2010, the patient suffered multiple reinterventions on the ADA and RCA for stent restenosis, followed by six more implantations of pharmacologically active stents.

At the current admission, we counted that 13 stents were implanted (11 of which were coronary stents), and a double aortocoronary bypass graft was performed.

Metoprolol succinate slow-release 50 mg, perindopril 5 mg, amiodarone 100 mg, clopidogrel 75 mg, aspirin 75 mg, atorvastatin 20 mg, and pantoprazole 40 mg are the drugs the patient takes on a daily basis.

The physical examination demonstrates a normoponderal status (BMI = 24 kg/m^2^) and a blood pressure of 160/100 mmHg with no detectable difference between the upper extremities. The results of the laboratory tests reveal that the patient has mild mixed dyslipidemia (low-density lipoprotein 61 mg/dL, high-density lipoprotein 46 mg/dL, total cholesterol 139 mg/dL, triglycerides 162 mg/dL) and mildly impaired renal function (creatinine 1.3 mg/dL, estimated glomerular filtration rate 58 mL/min/1.73 m^2^). Blood counts, serum electrolytes, fasting plasma glucose, glycated hemoglobin, liver and thyroid gland function tests, viral markers, and a coagulation profile were within normal limits. Cardiac enzymes were not raised (creatine kinase-myocardial band 19.6 U/L and troponin-I 0.40 ng/mL) and did not change during the admission.

**Figure 1:**
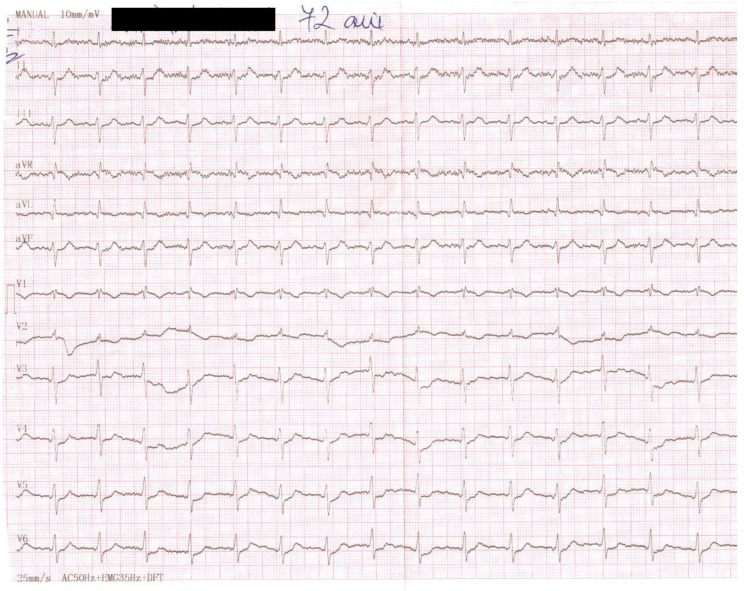
Admission electrocardiogram.

The ECG shows sinus rhythm, left axis deviation, a heart rate of 100 beats per minute, mild horizontal ST segment depressions in the DII, DIII, aVF, and V4-V6 leads, and a minor right bundle branch block (Figure 1).

**Figure 2:**
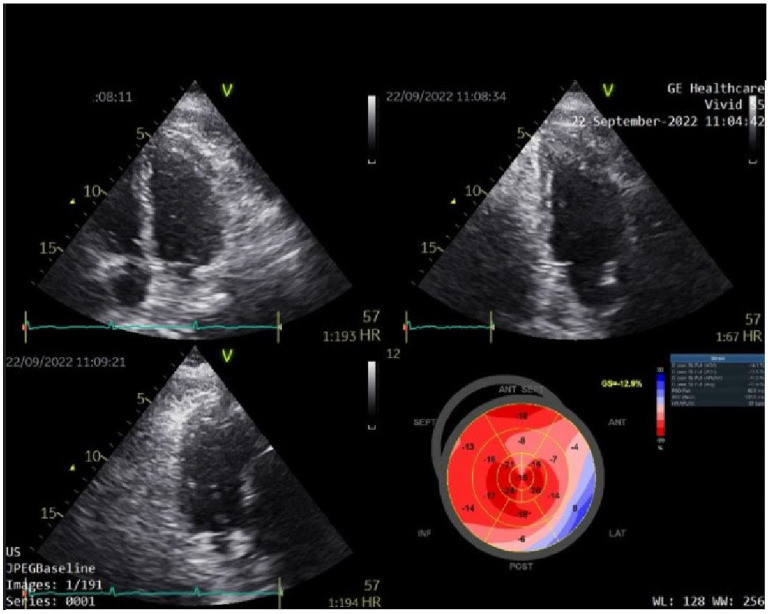
Two-dimensional echocardiography and Bull’s-eye map representation of global longitudinal strain of the left ventricle.

The echocardiographic measurements are normal, with a normal global systolic function and a left ventricular ejection fraction (LVEF) of 50%, but the LV wall motion is weak on the side (Figure 2).

**Figure 3:**
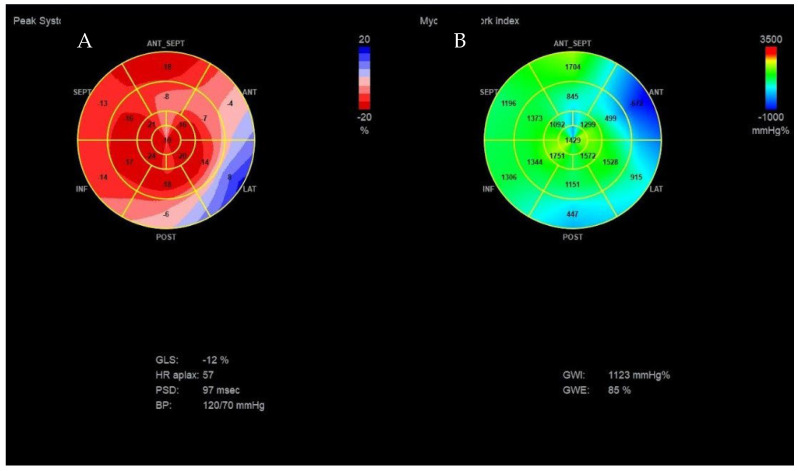
(**A**) Bull’s-eye polar map of left ventricular global longitudinal strain. (**B**) Myocardial work index, and efficiency.

We used speckle-tracking echography in 4-3- and 2-chamber apical incidences to calculate the global LV strain, which was found to be significantly decreased (<12%, normal range > 20%). We then assessed the myocardial work (MW) of the patient. The LV-pressure strain loop (PSL) in this new method is derived from non-invasively acquired brachial artery cuff pressure and generated by adjusting the profile of a reference LV pressure curve according to the duration of the isovolumic and ejection phases, as defined by the echocardiography timing of aortic and mitral valve events. After calculating the global LV strain, measurements of brachial blood pressure and time of valvular events were utilized to obtain the LV-PSL with currently available echocardiographic software. The area of the PSL represents approximately the MW and is computed by multiplying the rate of segmental shortening, obtained by integrating the strain curve, by the momentary LV pressure. This product is a measure of instantaneous power, which is incorporated throughout the cardiac cycle to generate MW, which is given in mmHg%. In addition to the MW index (work assessed from mitral valve closure to mitral valve opening), segments were examined for wasted work (WW) and constructive work (CW), with global values calculated as the mean of all segmental values. The global myocardial work index (1123 mmHg%; normal value > 1200 mmHg%), which measures LV work during systole, was reduced. As shown in Figure 3, myocardial work efficiency, which is calculated as the ratio of constructive to wasted work, was also found to be lower (85%, normal value > 90%).

The Bull’s-eye polar map representation of the global longitudinal strain of the LV, with 17 wall segments divided from apex to base and the visualization of a circumscribed blue area in specific segments, allows one to determine the distribution of blood flow abnormalities based on the culprit coronary artery.

On the polar map of the GLS, the regions with the lowest longitudinal strains were the anterior, lateral, and posterior ones, with the smallest value being observed at the level of the anterior region (−4%), as shown in Figure 3A.

The polar map of the global MW index (Figure 3B) also showed that the anterior, lateral, and posterior regions of the LV wall present lower values of the MW index, the lowest value being observed at the level of the posterior area (447 mmHg%).

**Figure 4:**
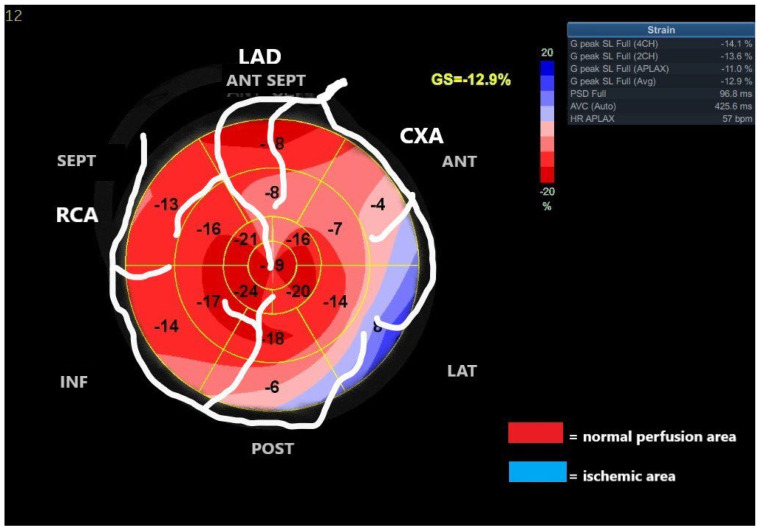
The Bull’s-eye polar map representation of regional global longitudinal strain based on a 17-segment model of distinct coronary artery vascularization areas.

The echographic images suggested severe blood flow abnormalities in these regions, which are irrigated from branches originating from the circumflex and from the right coronary artery, as shown in Figure 4.

**Figure 5:**
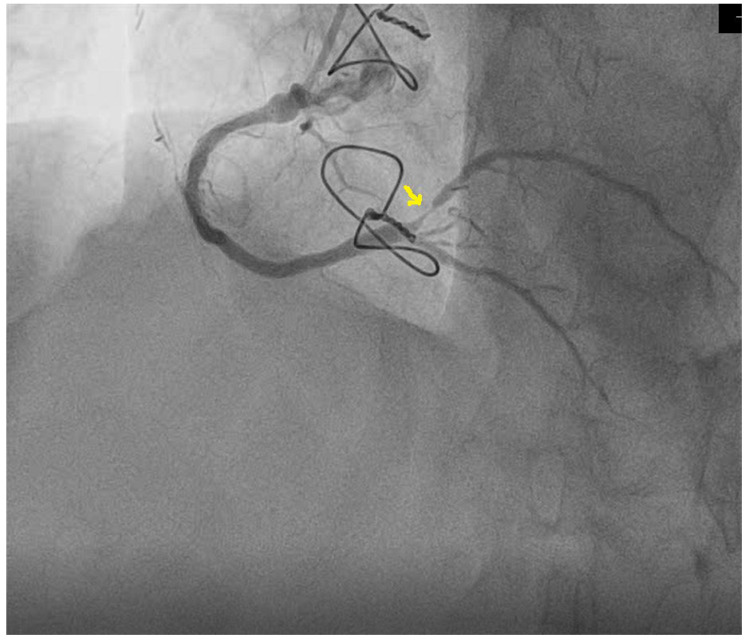
Sub-occlusive stenosis of the postero-lateral branch of the right coronary artery (arrow).

**Figure 6:**
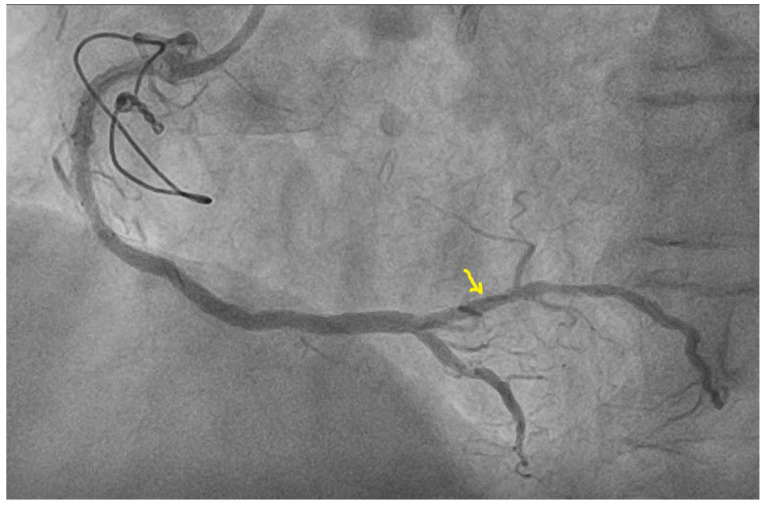
Coronary angiography post dilatation and stent implantation in the postero-lateral branch of the right coronary artery (arrow).

Considering the patient’s history, it was decided to perform coronary angiography, which showed multiple coronary stents, the right-dominant coronary system, and diffusely atheromatous coronary arteries; a LAD with ostial in-stent total chronic occlusion, but with a patent arterial graft on the mid segment; and an RCA with serial stenotic lesions of a maximum of 50–70% and a sub-occlusive lesion at the postero-lateral branch (PLB), as shown in Figure 5. Percutaneous coronary intervention was performed, and a 2.25–14 mm DES was implanted on the postero-lateral branch with a good final angiographic result (Figure 6).

The patient is discharged with complete angina remission. The medications recommended at discharge are: metoprolol slow release 100 mg daily; ticagrelor 180 mg daily; aspirin 75 mg daily; atorvastatin 80 mg daily; amiodarone 100 mg daily; amlodipine 5 mg daily; perindopril 5 mg daily; and pantoprazole 40 mg daily.

The long-term evolution has been favorable, with no angina five months after the last angioplasty.

## 3. Discussion

We are dealing with a hypertensive, smoking, and dyslipidemic patient with a history of two myocardial infarctions, 20 years apart, with multiple myocardial revascularization interventions over the course of 24 years, a carrier of 13 stents (11 coronary and 2 femoral) and of two aorto-coronary by-pass grafts.

The patient became very disciplined and compliant with medical indications after the first coronary event, but atheromatosis continued to progress, which raises the suspicion of a predisposing genetic factor. Also, multiple intrastent restenoses raise the suspicion of the development of resistance to antiproliferative agents in DESs.

PCI is a widely used technique for reopening coronary artery blockages [11]. In patients with a history of multiple myocardial revascularization interventions and ISR, it is difficult to differentiate de novo native coronary lesions from ISR. In-stent restenosis of drug-eluting stents, often known as “DES failure”, is one of the main reasons for recurrent target vessel revascularization and is linked to increased mortality and morbidity. ISR occurs more frequently in certain PCI patients, depending on patient and procedural factors, such as stent type, size, length, and number; lesion complexity (bifurcation, total occlusion, calcific lesions, etc.); the presence or absence of diabetes mellitus; etc. [12]. There is no consensus on the ideal therapeutic method for ISR management. The main options are the implantation of a new DES, treatment with a pharmacologically active balloon, CABG, or, in selected cases, only optimizing the treatment in order to control symptoms. As such, when these situations arise, they are difficult to evaluate and treat.

Individuals with persistent ISR typically have symptoms of stable angina or silent cardiac ischemia. They manifest clinically between three and six months after PCI [4]. Acute coronary syndrome (ACS) manifests as unstable angina in around 30% to 60% of ISR patients [4,13]. In almost 5% of instances, total vascular occlusion resulted in ST-elevation myocardial infarction [4,13].

In the case of BMS and DES, the underlying pathophysiology of ISR and its angiographic morphology might differ [12]. Nevertheless, the histological appearance of neointimal tissue biopsies from ISR locations in both kinds of stents is nearly the same. The occluding atheroma is mostly made up of lipids, proteoglycan-laden smooth muscle cells, and collagen and reticular fiber-rich sections [13]. Endothelial and smooth muscle cell proliferation, chronic inflammation of the vessel wall, macrophage activation, extracellular matrix deposition, progressive necrotic core formation, and intraplaque hemorrhages are some of the processes that eventually lead to atheroma formation [11,12].

Recoil and vessel dissection are the main reasons for failure with standard balloon angioplasty of the coronary vessels, with an incidence of 30% to 60%. Acute or chronic elastic recoil and negative constrictive remodeling of the vessel wall are the main processes at work [4,12,13]. Recoil does not occur when BMS is used. Neointimal hyperplasia, defined as the gradual and homogenous proliferation of smooth muscle cells, has been linked to lumen restenosis. It occurs early in the illness phase, followed by proliferative regression and angiographically detectable luminal expansion [14]. The prevalence of BMS-associated ISR ranges from 16% to 44%. Long lesion length and small vascular diameter are risk factors for BMS-associated ISR [13].

Antiproliferative agent resistance is an unusual cause of ISR. The cancer literature is well documented in terms of resistance to paclitaxel and sirolimus. The underlying process is represented by polymorphisms in the genes encoding mTOR or specific proteins in paclitaxel or sirolimus metabolism, resulting in reduced sirolimus binding to mTOR or FK-B12 [14,15]. This resistance may be genetically programmed or acquired during a cytotoxic response following medication exposure [16]. The persistence of the inflammatory response beyond 90 days following arterial injury is a secondary cause that may enhance the incidence of restenosis in a drug-resistant patient. The pattern of restenosis found in these cases is generally focal [17], and it occurs between 1 day and 90 days [16].

In terms of non-invasive prediction of coronary artery sub-occlusive lesions, longitudinal strain outperformed LVEF in identifying myocardial dysfunction induced by ischemia [18,19]. Recent research, however, has shown that LV myocardial strain is load-dependent. When the LV afterload was noticeably increased, the LV global strain dropped dramatically [20]. Non-invasive techniques independent of afterload have now been developed to detect myocardial dysfunction. Using speckle tracking and automated functional imaging analysis, global and regional myocardial work parameters can be easily determined. This procedure has the potential to be implemented in clinical practice and to add incremental value to the task of identifying high-risk ACS patients with unstable angina.

The regional MW index has been proven to be superior to all other echocardiographic indices (GLS, LVEF, etc.) in identifying critical coronary artery stenosis in acute coronary syndrome [8]. The presence of four adjacent segments with MW indexes of 1700 mmHg% demonstrated 81% sensitivity and 82% specificity in detecting coronary occlusion, with a negative predictive value of 94%, demonstrating superiority to the functional risk area measured with strain (sensitivity of 78%, specificity of 65%, and negative predictive value of 91%). GWI outperformed LVEF in diagnosing patients with critical coronary stenosis (sensitivity of 70 vs. 63%, specificity of 82 vs. 62%, and negative predictive value of 91 vs. 86%, respectively) [21]. 

Particularly in patients with multi-vascular coronary artery disease, the critical ischemia region may be difficult to identify by non-invasive methods. In our patient, LV longitudinal strain and myocardial work imaging detected severe abnormal deformation patterns of the anterior, posterior, and lateral regions of the LV wall, suggesting severe blood flow abnormalities in these segments. The region with critical ischemia was correctly identified by the regional MW index, showing the superiority of this new method compared to the LV regional longitudinal strain.

Urgent coronary angiography was performed, followed by successful myocardial revascularization.

## 4. Conclusions

In patients with a history of multiple myocardial revascularization interventions and ISR, it is difficult to differentiate de novo native coronary lesions from ISR. Myocardial work imaging was beneficial in the detection of the altered deformation patterns indicating significant ischemia, even if the ECG presented minor changes and the cardiac enzyme levels remained normal. The myocardial region with critical ischemia was correctly identified by the regional MW index, which proved superior to the regional LV strain. Urgent coronary angiography followed by angioplasty and stent implantation resolved the issue.

## Data Availability

The data supporting this study is with the author and has been included within the manuscript.
